# Should Bilateral Internal Thoracic Artery Grafting Be Used in Patients After Recent Myocardial Infarction?

**DOI:** 10.1161/JAHA.117.005951

**Published:** 2017-07-21

**Authors:** Dan Loberman, Dmitry Pevni, Rephael Mohr, Yosef Paz, Nahum Nesher, Mohamad Khaled Midlij, Yanai Ben‐Gal

**Affiliations:** ^1^ Department of Cardiothoracic Surgery Faculty of Medicine Tel Aviv Sourasky Medical Center Tel Aviv University Tel Aviv Israel; ^2^ Faculty of Medicine Tel Aviv University Tel Aviv Israel; ^3^ Division of Cardiac Surgery Brigham and Women's Hospital and Harvard Medical School Boston MA

**Keywords:** bilateral internal thoracic artery, BITA, myocardial infarction, Coronary Circulation, Ischemia, Revascularization, Cardiovascular Surgery

## Abstract

**Background:**

Bilateral internal thoracic artery grafting (BITA) is associated with improved survival. However, surgeons do not commonly use BITA in patients after myocardial infarction (MI) because survival is good with single internal thoracic artery grafting (SITA). We aimed to compare the outcomes of BITA with those of SITA and other approaches in patients with multivessel disease after recent MI.

**Methods and Results:**

In total, 938 patients with recent MI (<3 months) who underwent BITA between 1996 and 2011 were compared with 682 who underwent SITA. SITA patients were older and more likely to have comorbidities (diabetes mellitus, chronic obstructive pulmonary disease, chronic renal failure, peripheral vascular disease), to be female, and to have had a previous MI. Acute MI and 3‐vessel disease were more prevalent in the BITA group. Operative mortality of BITA patients was lower (3.0% versus 5.8%, *P*=0.01), and sternal infections and strokes were similar. Median follow‐up was 15.21 years (range: 0–21.25 years). Survival of BITA patients was better (70.3% versus 52.5%, *P*<0.001). Propensity score matching was used to account for differences in preoperative characteristics between groups. Overall, 551 matched pairs had similar preoperative characteristics. BITA was a predictor of better survival in the matched groups (hazard ratio: 0.679; *P*=0.002; Cox model). Adjusted survival of emergency BITA and SITA patients was similar (hazard ratio: 0.883; *P*=0.447); however, in the nonemergency group, BITA was a predictor of better survival (hazard ratio: 0.790; *P*=0.009; Cox model).

**Conclusions:**

This study suggests that survival is better with BITA compared with SITA in nonemergency cases after recent MI, with proper patient selection.

**Clinical Perspective**

**What Is New?**

- Left coronary system bilateral internal thoracic artery grafting (BITA) is associated with improved survival in patients undergoing coronary artery bypass grafting. The application of this complex technique in patients after acute myocardial infarction (MI) is controversial.
- In this study, we compared early and long‐term outcomes of patients with recent MI (<3 months) undergoing coronary artery bypass grafting with BITA versus single internal thoracic artery grafting.

**What Are the Clinical Implications?**

- Our analysis suggests that coronary artery bypass grafting with BITA may be suitable for patients operated on during the first week after a MI.
- Coronary artery bypass grafting with single internal thoracic artery grafting and vein grafts or radial artery is not inferior to BITA grafting in patients with recent MI.
- Selective use of BITA in nonemergency patients unmasks the benefits of BITA, and long‐term survival is better than that of nonemergency patients with single internal thoracic artery grafting.
- The results of this large observational study support the use of BITA in nonemergency patients after MI. Single internal thoracic artery grafting is a good alternative in emergency cases.

## Introduction

The efficiency of primary percutaneous intervention (PCI) and fibrinolysis in restoring myocardial blood supply and the longer time to surgery for patients with ST‐segment–elevation myocardial infarction (STEMI) “have resulted in less common use of coronary artery bypass grafting (CABG) as first line reperfusion strategy for patients with STEMI.”^1^

Furthermore, wide application of early PCI for patients with STEMI^2^, ^3^, ^4^, ^5^ has significantly decreased the number of emergency CABG procedures for myocardial revascularization. However, from 1996 to 2000 (the early years of the current report), surgical revascularization for reperfusion of patients with STEMI was more common.

Bilateral internal thoracic artery grafting (BITA) has been the preferred method of myocardial revascularization at the Department of Cardiothoracic Surgery, Tel Aviv Sourasky Medical Center, since 1996.^6^ Despite reported increased risk of sternal dehiscence and sternal infection,^7^, ^8^, ^9^ recent myocardial infarction (MI) was not a contraindication for using BITA between 1996 and 2011, the study period of the current investigation,. The purpose of this report is to compare, for the subset of patients with multivessel disease and recent MI (3 months), early and long‐term outcomes of BITA with those of CABG using single internal thoracic artery grafting (SITA) and other conduits such as saphenous vein grafts (SVGs) or the radial artery (RA). A secondary objective of this study is to compare the outcomes of BITA and SITA patients who underwent CABG <24 and >24 hours after MI.

## Patients and Methods

Data were obtained from review of medical records and telephone questionnaires. The study was approved by the institutional review board of the Tel Aviv Medical Center. The need for informed consent was waived by the institutional review board. Between 1996 and 2011, 3686 consecutive patients (3125 BITA and 561 SITA plus RA) with multivessel coronary artery disease underwent arterial revascularization at Tel Aviv Medical Center. They constituted 69% of primary CABG procedures for multivessel disease (5338 patients) performed at our institution during this time period. The remaining 1652 patients with multivessel coronary artery disease underwent SITA plus SVG.

We compared patient characteristics and procedure outcomes of 938 patients with multivessel coronary artery disease who underwent BITA within 3 months of STEMI with outcomes of 682 recent MI patients who underwent CABG with SITA and other conduits (eg, SVG or RA) at our center between 1996 and 2011. Follow‐up information was obtained by accessing data from the Israeli National Registry database.

During the study period, the selection of BITA or SITA grafting was made mainly according to the surgeon's preference. There was a tendency not to use BITA for patients with increased risk for sternal wound complications (older patients, patients with chronic obstructive pulmonary disease [COPD], or women with diabetes mellitus [DM] and/or obesity).^10^ In addition, the RA was used only when target coronary vessel stenosis was >80%.^11^, ^12^ All internal thoracic arteries (ITAs) were harvested as skeletonized vessels^13^ (Video S1).

Revascularization of the right coronary system was performed with SVGs, the right gastroepiploic artery, or the RA. In total 354 patients (21.9%) were operated without extracorporeal circulation.^13^, ^14^, ^15^ From the second postoperative day, RA and right gastroepiploic artery patients were treated with oral calcium channel blockers (diltiazem), 90 to 180 mg.^14^

### Definitions and Data Collection

Patient data were analyzed according to EuroSCORE I clinical data standards.^16^ DM was classified as non–insulin‐treated and insulin‐treated DM. Perioperative MI was defined as the postoperative appearance of new Q waves or ST‐segment elevation of >2 mm on an electrocardiograph, accompanied by a creatine phosphokinase myocardial band >50 mU/mL with or without a regional wall motion abnormality.^17^ A cerebrovascular accident was defined as a new permanent neurological deficit with computed tomographic evidence of cerebral infarction. Deep sternal wound infection (SWI) in this setting included patients with deep infection involving the sternum or substernal tissues in combination with patients with late dehiscence requiring sternectomy. Our definition of an emergency operation was based on the EuroSCORE and includes patients operated <24 hours of cardiac catheterization^16^ or those with ongoing angina, acute evolving MI, or pulmonary edema or in cardiogenic shock.^18^

### Statistical Analysis

#### Early outcome unmatched cohort

All data were summarized and displayed as mean±SD or median for continuous variables and as the number (percentage) of patients in each group for categorical variables. Categorical variables were compared using χ^2^ or Fisher exact tests, as appropriate. Continuous variables were compared using the independent‐sample *t* test or Mann–Whitney test.

Multivariate analysis of short‐term outcomes (operative mortality, sternal wound complications, perioperative MI, and stroke) was performed with binary logistic regression analysis. Adjusted odds ratios (ORs) and 95% confidence intervals (CIs) were reported. A propensity score was used to account for differences between groups in preoperative characteristics. The probability (propensity score) that a patient would receive SITA or undergo BITA according to preoperative variables was determined using a logistic regression model.^19^ Preoperative characteristics used for propensity score analysis were age, sex, non–insulin‐treated DM, insulin‐treated DM, DM with end‐organ damage, unstable angina, critical preoperative state,^16^ emergency operation, neurologic dysfunction, previous MI, acute MI (AMI), congestive heart failure, preoperative PCI, left main disease, number of diseased vessels, and left ventricular ejection fraction ≤30%. The type of conduit used (BITA or SITA) was forced into the multivariate models. We used the forward stepwise selection method to choose the predictors for inclusion in the multivariate analysis, which included the following preoperative and operative data: DM, peripheral vascular disease (PVD), cardiovascular disease, preoperative use of intra‐aortic balloon pump, repeat operation, chronic renal failure (CRF), left ventricular ejection fraction, number of grafts constructed, use of operative techniques such as operations performed without extracorporeal circulations (off‐pump coronary artery bypass), sequential grafting, and the use of other conduits such as RA, SVG or right gastroepiploic artery. The operative era (1996–2000 versus 2001–2011) was forced into regressions.

#### Late‐outcome unmatched cohort

Follow‐up, which was 97.3% complete, was obtained using the Israeli National Registry database. The log‐rank test and Kaplan–Meier curves were used to compare survival among groups.

A stratified Cox model was used to identify predictors of decreased survival. The propensity score quintiles were used for stratification. Adjusted hazard ratio (HR) and 95% CI were reported. The type of conduit used (BITA or SITA) was forced into the model. The forward stepwise selection method was used to choose the predictors for inclusion in the Cox model, and those were similar to the variables selected for the logistic regressions analyses.

#### Matching

Propensity score matching was used to generate a subcohort for further evaluation of early and long‐term outcomes. We accepted an absolute difference of up to 5% between propensity scores as a match.

The McNemar test and paired‐samples *t* test or Wilcoxon signed rank test were used to compare discrete and continuous variables, respectively.

#### Early outcome matched cohort

Multivariate conditional logistic regressions were performed to evaluate the association between the type of conduit used (group) and short‐ term outcomes.

#### Late‐outcome matched cohort

Kaplan–Meier curves were used to describe survival in the matched groups. Univariate and multivariate Cox regressions were used to compare long‐term outcomes among the matched groups. Variable selection in the multivariate analyses for the matched subcohort was performed as for the whole cohort.

The Hosmer and Lemeshow goodness‐of‐fit test was used to evaluate the logistic regression models. The likelihood ratio statistic, –2LL, and the overall χ^2^ were used to evaluate the Cox model. Moreover, the proportional hazards assumption was evaluated using the Schoenfeld residuals and the log minus log plot.

All multivariate regression included 4 blocks: The first included the type of conduit used, the second included age and sex using the enter method, the third included the preoperative and operative variables using the forward stepwise likelihood ratio method, and the fourth included the operative era and the propensity score (when it was part of the analysis) using the enter method.

The false discovery rate method was used to adjust the *P* values for multiple comparisons. All tests were 2‐tailed, and *P*<0.05 was considered significant.

Statistical analysis was performed with SPSS version 22 statistical software.

## Results

### Unmatched Results

Preoperative patient characteristics were significantly different between groups (Table 1). Patients treated with SITA were older and more likely to have comorbidities such as DM, COPD, CRF, and PVD; to be female; and to have had previous MI or a repeat or emergency operation. In contrast, AMI and 3‐vessel disease were more prevalent in the BITA group. The EuroSCORE of the SITA group was significantly higher than that of the group treated with BITA (Table 1).

**Table 1:** Patient Characteristics (Recent MI, N=1620)

Factor	Nonmatched BITA		Nonmatched SITA		*P* Value	Matched BITA		Matched SITA		*P* Value
No	938		682			551		551		
Age ≥70 y	340	36.2%	329	48.2%	0.001	243	44.1%	259	47.0%	0.780
Female	169	18.0%	200	29.3%	0.001	138	23.6%	122	22.1%	0.792
NIDDM	270	28.8%	282	41.3%	0.001	205	37.2%	203	36.8%	>0.999
IDDM	24	2.6%	51	7.5%	0.001	22	4%	17	3.1%	0.790
DM+EOD	60	6.4%	112	16.4%	0.001	57	10.3%	56	10.2%	>0.999
Congestive heart failure	286	20.9%	206	30.2%	0.043	175	31.8%	157	28.5%	0.780
Chronic renal failure	76	8.1%	97	14.2%	0.001	62	11.3%	73	13.2%	0.780
Peripheral vascular disease	162	17.1%	148	21.7%	0.006	101	18.3%	99	18.0%	>0.999
Cerebrovascular disease	76	8.1%	95	13.9%	0.001	51	9.3%	63	11.4%	0.780
COPD	52	5.5%	61	10.8%	0.001	48	8.7%	57	10.3%	0.780
EF ≤30%	95	10.1%	102	15.0%	0.002	68	12.3%	69	12.5%	>0.999
Unstable angina pectoris	351	37.4%	382	56.0%	0.001	250	45.4%	271	49.2%	0.780
ND	39	4.2%	54	7.9%	0.002	27	4.9%	32	5.8%	0.792
Previous MI >1 week	476	50.7%	372	54.5%	0.016	299	54.3%	294	53.4%	>0.999
Acute MI ≤1 week	590	62.9%	413	60.6%	0.029	326	59.2%	341	61.6%	>0.999
Left main disease	235	25.1%	186	27.3%	0.029	138	25.0%	152	27.6%	0.780
Three‐vessel disease	721	76.9%	495	72.6%	0.029	400	72.6%	410	74.4%	0.792
Critical preoperative state	118	12.6%	144	21.1%	0.001	94	17.1%	103	18.7%	0.792
S/P percutaneous intervention	139	14.8%	135	19.8%	0.003	92	16.7%	100	18.1%	0.792
Repeat operation	25	2.7%	25	3.7%	0.019	13	2.4%	19	3.4%	0.780
Emergency operation	198	21.1%	231	33.9%	0.001	147	26.7%	173	31.4%	0.780
EuroSCORE, mean±SD	6.87±3.43		9.50±4.33		0.001	7.85±3.56		8.85±4.11		0.360
OPCAB	179	19.1%	175	25.7%	0.001	118	21.4%	136	24.7%	0.780
Sequential grafts	443	47.2%	288	42.2%	0.008	248	45.0%	240	43.6%	0.853

BITA indicates bilateral internal thoracic artery grafting; COPD, chronic obstructive pulmonary disease; DM, diabetes mellitus; EF, ejection fraction; EOD, end‐organ damage; IDDM, insulin‐dependent diabetes mellitus; MI, myocardial infarction; ND, neurologic dysfunction; NIDDM, non–insulin‐dependent diabetes mellitus; OPCAB, off‐pump coronary artery bypass; SITA, single internal thoracic artery grafting; S/P, secondary or primary.

Operative mortality and occurrence of perioperative MI were lower in the BITA group (27 [3%] versus 31 [5.8%] patients in the BITA versus SITA groups, respectively; *P*=0.010 for operative mortality). Occurrences of SWI (23 [2.5%] versus 17 [2.5%] patients), perioperative MI (15 [1.6%] versus 18 [2.6%]), and stroke (23 [2.5%] versus 23 [3.4%]) did not reach statistical significance.

After forcing the propensity score and the operative period (OR: 1.8337 [95% CI, 0.737–2.424], *P*=0.339) into the logistic regression model, independent predictors of increased operative mortality were female sex (OR: 2.865 [95% CI, 1.629–5.050]; *P*<0.001), CRF (OR: 3.693 [95% CI, 1.985–6.871]; *P*<0.001); ejection fraction ≤30% (OR: 2.379 [95% CI, 1.314–4.307]; *P*=0.004), critical preoperative state (OR: 2.877 [95% CI, 1.222–6.776]; *P*=0.016), and preoperative use of intra‐aortic balloon pump. Off‐pump coronary artery bypass was associated with decreased operative mortality (OR: 0.449 [95% CI, 0.214–0.940]; *P*=0.034). The use of BITA was not a risk factor for operative mortality (OR: 0.876 [95% CI, 0.489–1.571]; *P*=0.657) or postoperative stroke (OR: 0.974 [95% CI, 0.508–1.873]; *P*=0.939); however, it was associated with increased risk of SWI (OR: 2.192 [95% CI, 1.016–4.729]; *P*=0.045).

Independent predictors of SWI were insulin‐treated DM (OR: 3.343 [95% CI, 1.188–9.409]; *P*=0.022), COPD (OR: 3.563 [95% CI, 1.616–7.855]; *P*=0.002), and repeat operations (OR: 4.379 [95% CI, 1.428–13.375]; *P*=0.010). Independent predictors of stroke were non–insulin‐treated DM (OR: 2.294 [95% CI, 1.130–4.650]; *P*=0.022), CRF (OR: 3.092 [95% CI, 1.477–6.471]; *P*=0.003), cardiovascular disease (OR: 1.896 [95% CI, 1.153–3.048; *P*<0.001), and preoperative cardiovascular disease (OR: 4.074 [95% CI, 1.987–8.355]; *P*<0.001).

**Figure 1:**
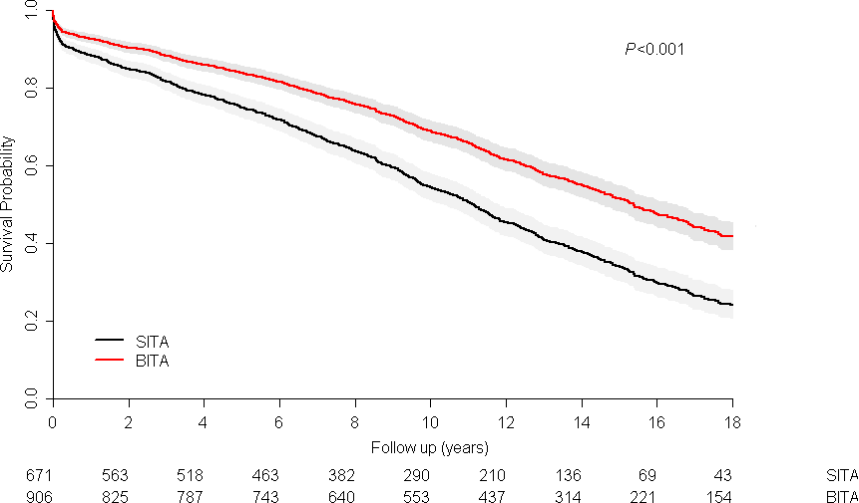
Kaplan–Meier survival curves of the bilateral internal thoracic artery grafting (BITA) and single internal thoracic artery grafting (SITA) groups before matching.

Follow‐up was 97.3% complete. The median follow‐up was 15.21 years (range: 0–21.25 years). The 10‐year survival (Kaplan–Meier) of the BITA group was significantly better than that of the SITA group (70.3±3.1% and 52.5±4.5%, respectively; *P*<0.001, log‐rank test; Figure 1) and assignment to the BITA group was associated with better survival (HR: 0.839 [95% CI, 0.718–0.981]; *P*=0.028) compared with the SITA group (Cox model; Table 2).

**Table 2:** Independent Predictors for Overall (Early and Late) Mortality^a^

	HR, 95% CI	*P* Value
Age	1.061, 1.053–1.069	<0.001
NIDDM	1.217, 1.045–1.417	0.011
DM+EOD	1.303, 1.024–1.660	0.032
COPD	1.570, 1.277–1.942	<0.001
CHF	1.178, 1.014–1.368	0.032
CRF	1.258, 1.008–1.570	0.042
Critical state	1.651, 1.375–1.984	<0.001
Repeat operation	1.486, 1.048–2.109	0.026
PVD	1.359, 1.149–1.607	<0.001
Threes‐vessel disease	1.241, 1.042–1.477	0.015
OPCAB	0.842, 0.708–1.002	0.053
1996–2000^b^	1.074, 0.915–1.260	0.374
BITA/SITA	0.839, 0.718–0.980	0.028

BITA indicates bilateral internal thoracic artery grafting; CHF, congestive heart failure; CI, confidence interval; COPD, chronic obstructive pulmonary disease; CRF chronic renal failure; DM, diabetes mellitus; EOD, end‐organ damage; HR, hazard ratio; IDDM, insulin‐treated diabetes mellitus; NIDDM, non–insulin‐treated diabetes mellitus; OPCAB, off‐pump coronary artery bypass; PVD, peripheral vascular disease; SITA, single internal thoracic artery grafting.

a Stratified Cox analysis.

b Early period vs 2001–2010.

### Matched Results

**Figure 2:**
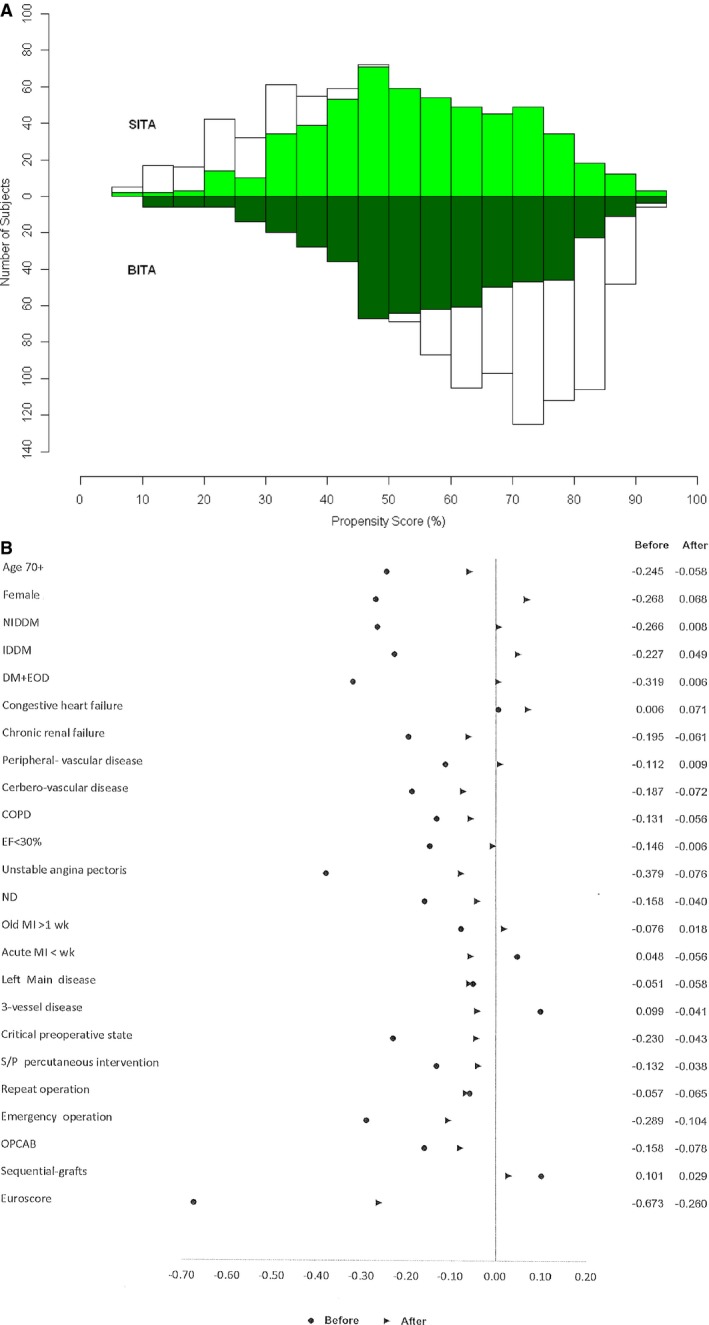
A, Mirrored histogram. B, Standardized difference. BITA indicates bilateral internal thoracic artery grafting; COPD, chronic obstructive pulmonary disease; DM, diabetes mellitus; EF, ejection fraction; EOD, end‐organ damage; IDDM, insulin‐dependent diabetes mellitus; MI, myocardial infarction; ND, neurologic dysfunction; NIDDM, non–insulin‐dependent diabetes mellitus; OPCAB, off‐pump coronary artery bypass; SITA, single internal thoracic artery grafting; S/P, secondary or primary.

To compare outcomes between groups, propensity score matching was performed; 551 pairs of well‐matched patients from the 2 groups were created (Table 1; Figure 2A and 2B).

Early mortality (30 days) was not significantly different between the 2 matched groups (5.1% and 3.4% for SITA and BITA, respectively; *P*=0.405). Furthermore, similar occurrences were observed for early postoperative SWI (1.3% versus 2.9%, *P*=0.375), perioperative MI (1.8% versus 1.8%, *P*<0.999), and postoperative stroke (3.4% versus 3.4%, *P*<0.999).

Kaplan–Meier curves for 10‐ and 15‐year survival of the matched BITA group were better (64.7±4.1% and 44.4±4.7%, respectively, for BITA versus 55.8±4.3% and 38.0±4.9%, respectively, for SITA; HR: 0.705 [95% CI, 0.574–0.865]; *P*=0.001; univariate Cox model). BITA also emerged as a predictor of better survival in multivariable analysis (HR: 0.679 [95% CI, 0.533–0.866]; *P*=0.002; Cox model).

PVD was a risk factor for decreased survival (HR: 1.693 [95% CI, 1.171–2.448]; *P*=0.005). Patients operated without off‐pump coronary artery bypass had better adjusted survival (HR: 0.646 [95% CI, 0.449–0.929]; *P*=0.018). Operative period (1996–2000 versus 2001–2011), which was forced into the model, was not a predictor of decreased survival (HR: 1.016 [95% CI, 0.722–1.430]; *P*=0.928).

### Results of Surgery Within 24 Hours Versus Later

To further evaluate our results, we divided the cohort to 2 subgroups according to operative timing (patients operated within 24 hours of admission [first day, emergency subgroup, 436 patients] versus those operated later [second or later day, nonemergency subgroup, 1183 patients]).

Operative mortality of BITA and SITA patients in the emergency subgroup was similar (15 [7.6%] versus 23 [10.0%] in the BITA and SITA groups, respectively; *P*=0.094).

In contrast, BITA patients in the nonemergency subgroup had significantly lower operative mortality (12 [1.6%] versus 18 [4.0%]; *P*=0.094).

**Figure 3:**
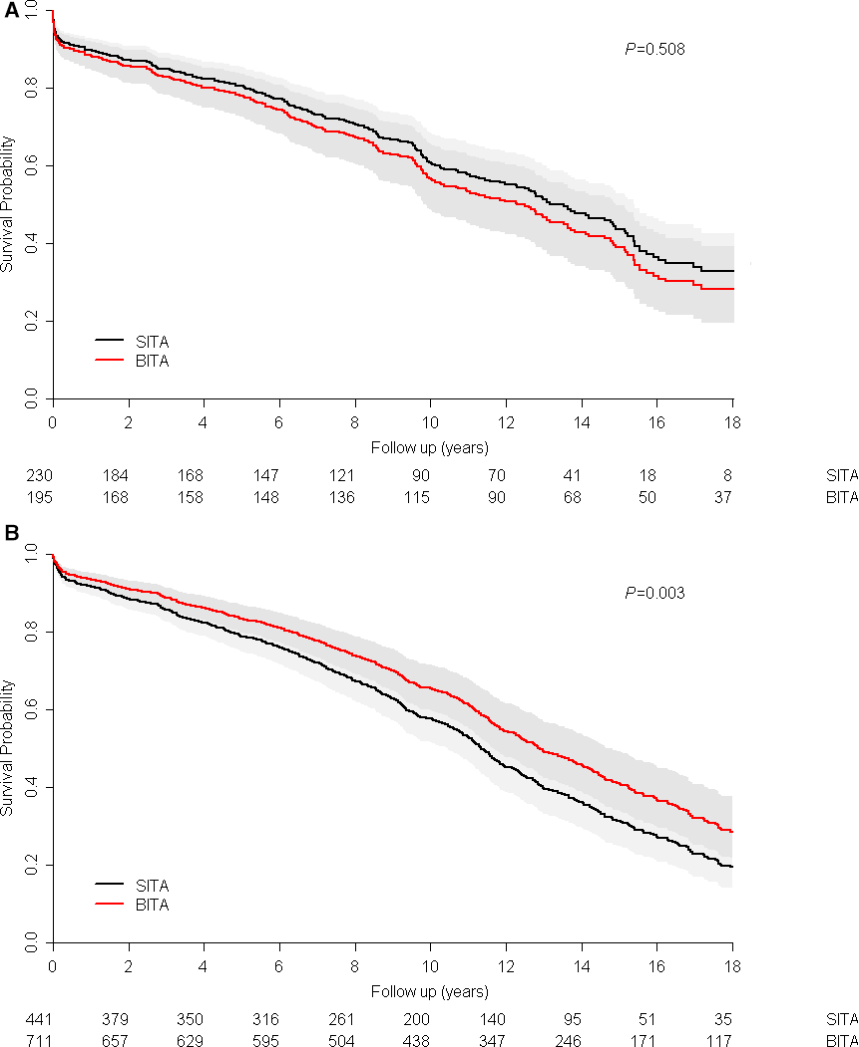
A, Kaplan–Meier survival curves of the emergency bilateral internal thoracic artery grafting (BITA) and single internal thoracic artery grafting (SITA) groups. B, Kaplan–Meier survival curves of the nonemergency BITA and SITA groups.

Kaplan–Meier survival curves for the BITA groups of both subsets were significantly better than those of the SITA groups. The probability (propensity score) that a patient would receive SITA or undergo BITA according to preoperative variables was determined for each subset by using a logistic regression model. We compared long‐term outcomes of BITA and SITA in the 2 subsets using a multivariate propensity‐stratified Cox model. Long‐term adjusted survival of BITA patients in the emergency subgroup was not better than that of SITA patients (Figure 3A; Table 3); however, in recent MI patients operated on ≥2 days after admission (nonemergency subgroup, Table 3), BITA was associated with significantly better long‐term survival (Figure 3B; Table 3).

**Table 3:** Predictors of Overall (Early and Late) Mortality According to Patient Urgency

	HR, 95% CI	*P* Value
Emergency (first day subgroup)		
BITA/SITA	0.883, 0.641–1.124	0.447
Age	1.064, 1.049–1.079	<0.001
NIDDM	1.387, 1.046–1.840	0.023
COPD	2.018, 1.375–2.962	<0.001
Critical state	1.739, 1.337–2.262	<0.001
Repeat operation	2.487, 1.240–4.988	0.010
Three‐vessel disease^a^	1.660, 1.201–2.294	0.002
rGEA	1.878, 1.087–3.245	0.024
1996–2000^b^	1.198, 0.882–1.628	0.247
Nonemergent patients (second and later day subgroup)		
BITA/SITA	0.790, 0.663–0.942	0.009
Age	1.066, 1.056–1.075	<0.001
DM+EOD	1.623, 1.281–2.056	<0.001
COPD	1.441, 1.117–1.859	0.005
CHF	1.379, 1.162–1.637	<0.001
Critical state	1.632, 1.192–2.236	0.002
PVD	1.382, 1.136–1.680	0.001
1996–2000^b^	1.067, 0.883–1.290	0.503

BITA indicates bilateral internal thoracic artery grafting; CHF, congestive heart failure; CI, confidence interval; COPD, chronic obstructive pulmonary disease; DM, diabetes mellitus; EOD, end‐organ damage; PVD, peripheral vascular disease; rGEA, right gastroepiploic artery; SITA, single internal thoracic artery grafting.

a Vs 2‐vessel disease.

b Vs 2001–2010.

## Discussion

Our study compared the early and long‐term clinical outcomes of CABG in patients with recent MI (3 months) and multivessel disease for those who underwent arterial revascularization using the skeletonized BITA technique and those who underwent CABG with SITA and other conduits such as SVG or RA.

The main finding in this report is the long‐term (median: 15.21 years), statistically significant survival benefit of BITA compared with SITA in nonemergency recent MI patients. This observation contrasts with the similar long‐term survival of SITA and BITA patients in the subset of emergency patients.

Several studies reported extensive arterial grafting with BITA as preferential treatment among selected groups, such as young male, nonobese, nondiabetic patients.^20^, ^21^, ^22^ In those studies, patients were preselected for BITA according to their life expectancy, and patients with comorbidities such as recent MI, DM, PVD, and age >70 years were not commonly offered BITA. In contrast, we did not preselect patients for BITA according to their comorbidities and life expectancy. Routine left‐sided arterial (left anterior descending and circumflex system) revascularization was the preferred method of revascularization at our center during the study period.^6^, ^13^ Thus, most (3683 patients, 69%) of the primary CABG procedures for multivessel disease that were performed at our institution during the study period were skeletonized BITA, and 938 underwent CABG within the first 3 months of AMI.

Two thirds of the patient cohort was from earlier years (1996–2000) and perhaps this explains why patients were operated on so early after infarction. Most surgeons today would agree that Q‐wave infarctions should not be operated within 24 hours unless other factors are present.

Selection of surgical approach for the current cohort was based on surgeons’ decisions. The main criterion considered for BITA was a reduced risk of deep sternal wound complications; high‐risk patients (those with COPD and obese and diabetic women) were preferentially referred to the SITA group during the study period.^10^ Consequently, patients treated with SITA were older; were more often female; and were more likely to have DM, COPD, CRF, and PVD and to have undergone a repeat or emergency operation. In contrast, AMI and 3‐vessel disease were more prevalent in the BITA group. The mean EuroSCORE of the SITA group was also significantly higher than that of the BITA group (9.94±4.46 versus 6.84±3.47, *P*<0.001).

Propensity matching was used to account for differences between groups in preoperative demographic and clinical characteristics. The risk profile of the matched groups was largely determined by the higher risk of the SITA group. This can explain the high occurrences of comorbidities and emergency operations and the relatively unfavorable long‐term outcomes of patients in our study compared with those in other BITA series.^13^, ^20^, ^21^, ^22^, ^23^, ^24^

Operative mortality in the BITA group was lower than in the SITA group (3.0% versus 5.8%, *P*=0.01); however, occurrences of early postoperative complications (SWI, perioperative MI, and postoperative stroke) did not differ significantly between the 2 groups. Moreover, the type of conduit that was used was not a significant predictor of operative mortality, SWI, perioperative MI, or stroke in multivariable analysis performed after forcing the propensity score and the operative period into the logistic regression models.

This similar early outcome justifies the surgeons’ preferences and patient selection for BITA or SITA based on the risk for SWI.

Despite the use of skeletonized ITAs,^25^ the rate of SWI was relatively high. This may be explained in part by the nonselective use of BITA and by the fact that precautions such as preoperative nasal swabs, nasal disinfection, strict postoperative glucose control, and enhanced sternal wound stabilization were not yet employed during the early years of the study (1996–2000). Increased prevalence of cardiovascular disease, PVD, non–insulin‐treated DM, and neurologic dysfunction and the fact that a significant proportion of the BITA patients were operated in this early era (before introduction of epiaortic ultrasound and preoperative computed tomography angiography for routine use, and before popularization of the aortic no‐touch off‐pump coronary artery bypass technique) can also explain the relatively high occurrence of strokes.

CABG is currently reserved mostly for AMI patients after unsuccessful or complicated primary PCI^2^, ^3^ and for unstable MI patients with left main or severe 3‐vessel disease who cannot be treated safely with PCI.^4^ CABG is also recommended for patients with late presentation of STEMI (>12 hours [late arrivals]) who are asymptomatic. For these patients, the American College of Cardiology and American Heart Association guidelines for CABG and PCI are similar to those for patients with stable coronary artery disease.^4^ Similar management is recommended for patients after primary PCI who develop recurrent angina or reinfarction.^5^

Unlike the current practice that reserves CABG mostly for complicated or unsuccessful primary PCI, during the early years of this report (1996–2000), surgical revascularization (including BITA) for reperfusion of MI patients was more commonly used.

In a previous publication from our center, Nesher et al showed that long‐term outcomes of patients who underwent arterial grafting within the first 7 days of an AMI (including emergency and intra‐aortic balloon pump–supported patients) were similar to those of patients with AMI operated later. This was despite their increased operative mortality.^26^ The long‐term results of the current report suggest that arterial grafting not only is the preferred approach for patients with stable angina but also can be suitable for patients operated during the first week after AMI.

A number of studies have shown that the use of an additive right ITA to the left ITA improved survival.^13^, ^21^, ^23^, ^24^, ^27^ In their large study evaluating the patency rate of the various conduits used for myocardial revascularization, Tatoulis and colleagues reported an excellent patency rate for the right ITA, which was equivalent to that of the left ITA for identical territories. The patency rate of the right ITA was better than those of RA and SVGs. This better patency rate of BITA was associated with improved long‐term survival.^23^ Our results are not supported by a recently published propensity score–matched study from our center that showed BITA is not better than SITA and RA when the RA was connected end to side to the left ITA as a composite T graft.^28^

Our findings are strongly supported by a large landmark meta‐analysis performed in 2001 by Taggart and colleagues, who demonstrated significantly better survival for BITA than SITA patients.^22^ Further support for this observation is evident from large studies of diabetic patients with longer mean follow‐up^27^, ^29^ and from another study that showed the right ITA to be better than the RA as a second arterial conduit.^24^

The better long‐term survival of BITA patients in the above reports, as in our report, was further confirmed by multivariable Cox analysis, which showed BITA to be associated with better long‐term survival in both unmatched and propensity score–matched groups of patients. Despite the differences in patient selection between the above studies and the current report (the BITA patients in our study were at significantly higher risk [acutely ill with recent MI and other co‐morbidities] and older), long‐term results are similar. The effect of age and, more important, comorbidities on late mortality can explain the forcing out of BITA/SITA assignment from the propensity‐adjusted Cox models in the subset of emergency patients in our study.

The single‐center observational retrospective design of our study presents limitations. Complete postoperative angiographic data and complete follow‐up clinical data were not available for all major adverse cardiac events; therefore, end points such as late MI, cardiac mortality, and reinterventions that were not collected prospectively were not complete, and their occurrences could not be compared between groups. Consequently, major adverse cardiac events were not assessed in the analysis. Given the sample size of the matched groups, it is likely that event‐free survival may have revealed differences between groups.

Another limitation is the possible selection bias in the criteria used for the choice of the second conduit and the tendency of surgeons not to use BITA in patients with increased risk for sternal wound complications (eg, COPD, obese diabetic women). Therefore, patients treated with SITA were older and higher risk. This surgeon selection bias is only partly accounted for in the propensity adjustment procedure. It may cause bias, even though matching has been performed, and modify the results.

In conclusion, CABG with SITA and SVGs or RA is not inferior to BITA in patients with recent MI. Earlier mortality from noncardiac causes reduces contribution of the type of conduit used and increases the influence of competing preoperative comorbidities on propensity‐adjusted survival (Cox model). However, selective use of BITA in nonemergency patients unmasks the benefits of BITA, and long term survival is better than that of nonemergency SITA patients. The results of this large observational study support the use of BITA in nonemergency patients after MI. SITA is a good alternative in emergency cases.

## Disclosures

None.

## Supporting information


**Video S1.** BITA harvest and skeletonization. Best viewed with Windows Media Player.
